# Piloting a Digital App Based on the Friendship Bench for Depression and Anxiety in Palestine: Mixed Methods Study

**DOI:** 10.2196/77507

**Published:** 2026-08-05

**Authors:** Chantal Lakis, Jamilah Sherally, Anne Braakman, Shruthi Abirami Ramiah, Maarten van Herpen, Sireen Khammash, Luma Tarazi, Umaiyeh Khammash, Jennifer Dabis, Elaine Rabello, Mahdi Adelwahab, Pierre Pratley, Dixon Chibanda

**Affiliations:** 1 Global Health Unit, KIT Institute, Royal Tropical Institute, Mauritskade 64, Amsterdam, 1092 AD, The Netherlands, 31 0205688711; 2 InukaCare B.V, Almere, The Netherlands; 3 Mental Health Services, Juzoor for Health and Social Development, Ramallah, Occupied Palestinian Territory; 4 Faculty of Epidemiology and Population Health, London School of Hygiene & Tropical Medicine, London, United Kingdom

**Keywords:** digital mental health, depression, posttraumatic stress disorder, conflict, task shifting, lay workers, coaching

## Abstract

**Background:**

The burden of mental disorders is high in conflict-affected populations. In Palestine, we piloted Inuka Coaching, a digital intervention adapted from the Friendship Bench delivered by trained and supervised lay coaches. This paper documents the implementation of the intervention in this highly volatile context after October 7, 2023.

**Objective:**

This study aimed to describe the implementation of Inuka Coaching, a digital mental health tool based on task shifting, in Palestine and examine contextual challenges, fidelity to the coaching model, and lessons learned regarding recruitment, retention, and delivery during escalating ethnic cleansing.

**Methods:**

Two Palestinian mental health professionals were trained and certified in the Inuka method as head coaches, and subsequently trained 5 lay coaches. Palestinian adults in Gaza and the West Bank were recruited primarily through social media and received up to 4 structured, text-based coaching sessions typically delivered over 4 weeks depending on participant availability and preference. Standardized mental health screening questionnaires (Self-Reporting Questionnaire–20 [SRQ-20] and Posttraumatic Stress Disorder Checklist for the *Diagnostic and Statistical Manual of Mental Disorders, Fifth Edition* [PCL-5]) were collected at baseline, immediately after the first coaching session, and 3 months after the final session. Session transcripts were reviewed to assess coaches’ fidelity to the Inuka method, and a focus group discussion explored coaches’ experiences with training, delivery, and contextual challenges.

**Results:**

Between August 2023 and February 2024, a total of 70 participants were enrolled. Baseline assessments indicated high levels of psychological distress: 95.7% (67/70) scored above the PCL-5 threshold of 31, suggesting likely posttraumatic stress disorder, and 69.4% (43/62) scored in the “at risk” range on the SRQ-20. The effectiveness of the method could not be determined as retention was low, with only 7.1% (5/70) completing the program. Coach fidelity was high, with 94.6% (35/37) of transcripts adhering to all 5 steps of the intervention. Coaches reported positive experiences with the method but identified challenges related to recruitment, session continuity, platform usability, and the need for flexibility during acute crises. Key implementation learnings included the importance of early in-person collaboration and training, culturally sensitive framing, flexible delivery and session structures, and robust support for lay coaches.

**Conclusions:**

While digital, task-shifted mental health interventions can be delivered with fidelity in conflict settings, sustaining engagement during escalating violence remains challenging. Future implementations require flexible design, context-sensitive recruitment and retention strategies, adaptive delivery models, and strong support for lay coaches.

## Introduction

Mental health conditions affect millions worldwide, causing significant disability and high economic and social costs [1]. The burden is particularly high in conflict-affected populations, with estimates from a 2019 systematic review and meta-analysis indicating that more than 1 in 5 people (22.1%, 95% uncertainty interval 18.8%-25.7%) experience mental disorders such as depression, anxiety, posttraumatic stress disorder (PTSD), bipolar disorder, or schizophrenia [2]. Additionally, the prevalence of depression and anxiety in these settings is approximately 5 times higher than the global average at 10.8% and 21.7%, respectively [2].

To achieve equitable global mental health, “task sharing” and community-based interventions have been identified as key innovations to be promoted and prioritized [1]. Growing evidence shows that nonprofessionals or lay coaches ease the burden on the health system and can effectively provide counseling for common mental health disorders (CMDs) such as depression and anxiety after receiving training from professionals [3,4]. These coaches can be recruited from the communities they serve and may even be peers [3]. Studies in low- and middle-income countries have found that such interventions have the potential to reduce symptoms of trauma, depression, and anxiety [4]. An example is Zimbabwe’s Friendship Bench, where community grandmothers were trained to deliver problem-solving therapy for CMDs [5]. In a randomized controlled trial in Zimbabwe with 573 adults screening positive for CMDs, the intervention led to significant improvements in primary CMD outcomes (measured using the Shona Symptom Questionnaire), as well as reductions in secondary depressive symptoms (measured using the Patient Health Questionnaire) at 6 months [5].

Inuka Coaching is a digital version of the Friendship Bench method and has been piloted in Kenya [6] and Zimbabwe [7]. Clients first take a 20-question scan, the Self-Reporting Questionnaire–20 (SRQ-20), and are subsequently linked to a certified coach for text or video sessions. The SRQ-20 is a tool developed by the World Health Organization to screen for CMDs and consists of 20 brief “yes” or “no” questions assessing symptoms of anxiety, depression, and somatic distress experienced over the preceding month [8]. Each Inuka Coaching session begins with active listening, where clients identify their concerns, select a primary issue to address, brainstorm solutions, and then collaboratively develop an action plan for follow-up. The coach refers clients needing specialized support to mental health professionals. Session quality is ensured through a structured quality assurance process that includes regular transcript reviews by certified psychiatrists, coach performance ratings, and the use of pre- and postintervention assessments to monitor client progress.

While Inuka Coaching has expanded to more countries in Africa and Europe, it has not yet been tested in the Middle East and North Africa region. In addition, there is limited literature on digital mental health solutions delivered through task sharing in conflict-affected settings. Digital interventions have been researched in Iraq [9] and Lebanon [10], as have lay counselor–led interventions in Iraq [11]. However, to our knowledge, only 2 studies conducted in Lebanon have examined the effectiveness and acceptability of combining these approaches [10,12]. In line with this, a recent systematic review of mental health apps for underserved populations worldwide concluded that few digital tools have been tailored to meet context-specific needs, and more rigorous research on acceptability, effectiveness, and cost-effectiveness is required before mental health apps are scaled up [13].

In Palestine, prolonged exposure to persecution, violence, displacement, and economic hardships has resulted in some of the highest mental health burdens in the region [14,15]. At the time of writing, civilians in the Gaza Strip lack access to basic humanitarian and medical services, with international aid agencies desperately calling for a ceasefire. Despite the high need, the country’s mental health care capacity is limited, and access is hampered by poor infrastructure, restricted freedom of movement, and pervasive stigma [15,16]. A study in the West Bank showed that digital interventions have the potential to offer widespread solutions, with 93.4% of residents having access to a phone and most (88.2%) convinced of the value of mobile interventions to people with mental health problems [17]. Through combining task shifting with a digital mode of service delivery, we hypothesized that barriers currently preventing Palestinians from seeking help for their mental health problems could be overcome. This paper describes the piloting of Inuka Coaching in Palestine and examines baseline mental health needs, intervention delivery and acceptability, coach fidelity, and implementation challenges in a context of escalating violence. In doing so, this study sought to generate practice-based insights to inform the future design and implementation of digital mental health interventions in conflict-affected contexts.

## Methods

### Study Design

Our pilot study used a mixed methods approach. Participants’ pre- and postintervention scores on the SRQ-20 and PTSD Checklist for the *Diagnostic and Statistical Manual of Mental Disorders, Fifth Edition* (PCL-5), were used to assess the preliminary effectiveness of Inuka Coaching in reducing CMD symptoms. To evaluate coaches’ fidelity to the method, session transcripts were systematically scored against established criteria. Additionally, we conducted a focus group discussion with coaches to explore experiences with the method and its contextual application.

### Setting

This study was conducted between August 2023 and February 2024 in Gaza and the West Bank. It was a collaboration among Juzoor for Health and Social Development, Inuka Coaching, and the KIT Institute. Juzoor for Health and Social Development (hereafter referred to as “Juzoor”) is a Palestinian nongovernmental organization established in 1996 that promotes the health and well-being of Palestinian communities through a multi-sectoral, rights-based development approach aligned with national priorities. Juzoor and the KIT Institute have collaborated on multiple projects since 2017. Inuka Coaching has previously delivered digital mental health services to employees of the KIT Institute.

Building on these existing partnerships, the 3 organizations jointly participated in a Dutch government Small Business Innovation Research program. This program is an innovation competition through which the government challenges entrepreneurs to develop and test solution-oriented products addressing societal challenges. Within this pilot, Juzoor served as the local implementing partner, providing access to lay coaches, referral networks, and contextual expertise; the KIT Institute acted as the knowledge partner and coordinated the overall process; and Inuka Coaching functioned as the social enterprise and technical innovator, providing training, supervision, and technical support.

### Recruitment

#### Coaches

Two Palestinian mental health professionals employed by Juzoor (SK and LT) were recruited as head coaches and trained in the Inuka method through online certification by the Inuka team. The training covered the core principles of the intervention, session structure, referral criteria, and the use of the digital platform. Certification was granted after the head coach had completed practice sessions. Subsequently, 5 laypersons from Juzoor’s network were similarly trained and certified by the head coaches using a train-the-trainer model. Lay coaches were also trained to identify participants requiring escalation to head coaches, and an additional pathway was established for external referrals to specialized clinical care. For quality assurance, the head coaches provided ongoing supportive supervision and conducted regular transcript reviews. Head coaches delivered their supervisory roles as part of their regular employment with Juzoor, whereas lay coaches received financial compensation per session delivered. In addition, 2 Juzoor staff members supported the intervention by providing customer support for the digital platform.

Prior to participant recruitment, the study team ran a preparatory pilot in which head coaches, lay coaches, and customer service personnel practiced study procedures. This allowed for refinement of workflows and procedural adjustments before full study commencement.

#### Participants

Eligible participants were adults aged 18 years and older residing in Gaza or the West Bank who could independently use a smartphone. Exclusion criteria were suicide risk (question 17 on the SRQ-20) and SRQ-20 scores above 12, necessitating referral to professional mental health services. Due to budgetary constraints, we set a target sample size of 100 participants, aiming for diversity in gender, age, and residence. Recruitment strategies included word of mouth and partnerships with community-based organizations, which distributed culturally adapted communication materials (Multimedia Appendix 1). These materials were posted on social media (Facebook and Instagram) and displayed at local community centers. Additional recruitment efforts included collaborating with universities; engaging youth ambassadors; and leveraging local media, including radio spots and partnerships with influencers, to broaden outreach (Multimedia Appendix 2).

### Data Collection

The SRQ-20 was used to assess symptoms of CMDs and was selected due to its validation and widespread use in low-resource settings. The PCL-5 was included to capture trauma-related symptoms given the conflict context. As no validated Arabic versions of the SRQ-20 or PCL-5 were available, we translated them, as well as the digital Inuka platform, into Arabic, adapting them to the Palestinian context, and conducted a back translation into English to ensure accuracy. Upon initial phone contact, potential participants received study details from customer service personnel, confirming voluntary participation, confidentiality, secure data handling, and the option to withdraw at any time. No financial incentives were offered. Those who verbally consented to participate received a WhatsApp link to complete the SRQ-20. Eligible clients were then matched with a coach. Customer service maintained a confidential file linking participant names to anonymous IDs accessible only to head coaches if safety concerns were identified. No information on previous mental illness or use of mental health services was collected. The Inuka Coaching method entailed receiving four 30- to 45-minute text-based weekly sessions via WhatsApp. While the method could be delivered via videoconference, we opted for text due to anonymity and internet connectivity (eg, in Gaza, for much of the study period, only 1G networks were available). Sessions followed a structured format, using predefined steps and prompts rather than a fixed curriculum. Each session started with active listening, after which participants identified and prioritized current concerns, collaboratively brainstormed potential solutions, and selected feasible actions. These actions were formulated as specific, measurable, attainable, relevant, and time-bound goals and documented in a personalized well-being plan, which was revisited and updated in subsequent sessions. While a reduction in psychological distress was an intended outcome, the primary aim of the intervention was to strengthen participants’ problem-solving skills and coping strategies.

The SRQ-20 and PCL-5 scales were administered at 3 time points: baseline (T1), immediately following the final session (T2), and 3 months after T2 (T3). Transcripts of the sessions were anonymously documented on the Inuka platform.

To assess fidelity to the Inuka method, a scoring system was developed based on the method’s 5 steps. Anonymized session transcripts were reviewed and scored by 3 external assessors (CL, JS, and ER), with adherence to each step documented as present or absent. When steps were not completed, the reviewer could insert free text explaining the context. A transcript was considered compliant with the Inuka Coaching method when all 5 steps were adhered to.

Coaches’ perspectives on the method and platform were collected through an online focus group discussion conducted by an Arabic-speaking researcher (MA) external to the pilot yet familiar with the study context and intervention. The topic guide covered user engagement, usability, effectiveness, accessibility, feedback, privacy, perceived user satisfaction, and observed behavior changes, as well as the impact of the war on coaches’ use and/or experience of the app (Multimedia Appendix 3). The group discussion was recorded for analysis.

### Analysis

#### Effectiveness

Quantitative analysis was planned on SRQ-20 and PCL-5 scores across the 3 time points in Stata (version 15; StataCorp). For the SRQ-20, scores between 0 and 6 were categorized as “resilient,” scores between 7 and 16 were categorized as “at risk,” and scores of 17 and above were categorized as “in a tough place.” For the PCL-5, a cutoff score of 31 was considered indicative of PTSD [18]. Mean differences in SRQ-20 and PCL-5 scores were analyzed to evaluate changes in CMD symptoms over time. Descriptive statistics summarized sociodemographic characteristics to contextualize the findings.

#### Fidelity

Fidelity scores were calculated as the percentage of steps completed. The free-text responses were analyzed thematically, enabling the identification of categories of nonadherence.

The five questions evaluated were (1) did the coach facilitate listing or eliciting problems of the client? (2) Did the coach facilitate choosing one problem to work on and understand better? (3) Did the coach facilitate formulating a goal? (4) Did the coach facilitate brainstorming toward solutions? (5) Were one or more of these solutions put into specific, measurable, attainable, relevant, and time-bound action steps?

#### Acceptability

Findings from the recorded focus group discussion were documented in English by MA using a rapid content analysis approach that used a matrix to organize responses across key themes from the topic guide. Following initial documentation, MA synthesized the findings into a structured summary report, which was shared with the wider research team. The report was reviewed and discussed collectively to refine interpretation and resolve ambiguities, with CL and JS going back to the raw data when needed.

### Ethical Considerations

Ethics approval was secured from the Palestinian Health Research Council under reference number PHRC/HC/1054/22. Informed consent was obtained from all participants prior to enrollment. The consent form outlined the study’s purpose, procedures, potential benefits, and risks, and explicitly addressed participant privacy and confidentiality. All recorded information was anonymized. Participant names were recorded only once, on a password-protected document accessible solely to the study coordinator, to be used only in case of emergency. Any information shared beyond the immediate study team was presented exclusively in aggregate form, with no individual-level data disclosed. Participants were also informed of their right to withdraw at any time without consequence to their access to community-based organization services.

## Results

### Participant Demographics, Completed Sessions, and Main Outcomes

Recruitment commenced in August 2023 but slowed down considerably due to the escalating violence in Gaza after October 7, 2023. In total, 70 participants were enrolled, 65.7% (n=46) of whom joined through social media channels. Most participants were under 30 years of age (n=47, 67.1%), women (n=57, 81.4%), and based in the West Bank (n=56, 80%; Table 1). We did not collect information on the use of any alternative formal mental health support.

**Figure 1.**
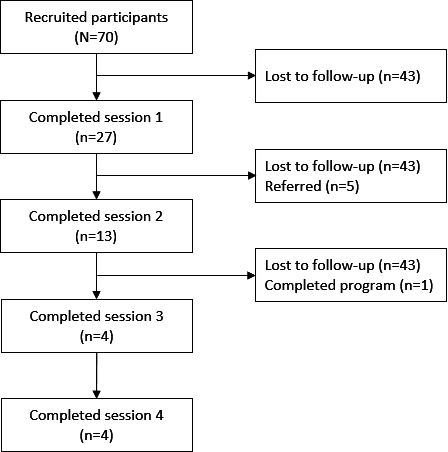
Participant recruitment and loss to follow-up throughout the study period.

Of the 70 enrolled participants, 5 (7.1%) completed all the program, 5 (7.1%) required referral to specialized services, and 1 (1.4%) ended the program after 2 sessions following mutual agreement with the coach. Attrition was highest in the early stages of the program. Of the 27 (38.5%) remaining participants, 33.3% (9/27) were lost to follow-up and 18.5% (5/27) were referred to more specialized care after the first session. The remaining 13 participants completed session 2, of whom one was discharged by the coach and considered to have completed the program, while 61.5% (8/13) were lost to follow-up. The remaining 4 participants completed sessions 3 and 4 (Figure 1). In summary, 5 participants were considered to have completed the program: 1 with 2 sessions and 4 with 4 sessions.

The SRQ-20 was collected through a link sent to participants, whereas the PCL-5 was collected by customer care staff at the time of participant registration. SRQ-20 scores for 88.6% (62/70) of the clients and PCL-5 scores for 100% (70/70) of the clients were collected at intake: 69.4% (43/62) scored as “at risk” on the SRQ-20, and 25.8% (16/62) were categorized as being “in a tough place.” For the PCL-5, a total of 95.7% (67/70) scored above 31, suggestive of PTSD. The 12.9% (8/62) of missing SRQ-20 scores were due to participants not completing the link that was sent to them.

Of the 5 participants who completed all 4 coaching sessions, 4 had both pre- and postintervention SRQ-20 data, and all 5 had pre- and postintervention PCL-5 data. On the SRQ-20, a total of 3 of the 4 participants showed a reduction in symptom scores and were classified as “resilient” at program completion. On the PCL-5, all 5 participants demonstrated reduced symptom scores, with a median reduction of 14 (IQR 13.5-17.5) points. Given the small number of completers and the case study design, these changes are presented descriptively and cannot be attributed to the intervention alone.

**Table 1.** Sociodemographic characteristics of participants who reached out through the Inuka platform (N=70).

Characteristic	Participants, n (%)
Age	
Early adulthood (≤30 y)	47 (67.1)
Middle adulthood (31-59 y)	22 (31.4)
Older adulthood (≥60 y)	1 (1.4)
Gender	
Women	57 (81.4)
Men	13 (18.6)
Marital status	
Never married	37 (52.9)
Married	25 (35.7)
Divorced or separated	6 (8.6)
Widowed	2 (2.9)
Employment status	
Unemployed	38 (54.3)
Employed full time	25 (35.7)
Employed part time	6 (8.6)
Daily worker	1 (1.4)
Disability (no)	68 (97.1)
Chronic illness (no)	66 (94.3)
Location	
West Bank	56 (80)
Gaza	14 (20)
Type of location	
Urban	49 (70)
Rural	16 (22.9)
Camp	5 (7.1)
Recruitment channel	
Social media	46 (65.7)
Word of mouth	18 (25.7)
Poster	6 (8.6)

### Coach Fidelity

A total of 57 transcripts were reviewed to evaluate the fidelity of the coaches to the Inuka method. Of these 57 transcripts, 20 (35.1%) could not be evaluated primarily due to 3 factors: participants ceasing to respond midsession (n=9, 45%), time constraints resulting in the next steps being deferred to a subsequent session (n=4, 20%), participants requiring referral (n=3, 15%), or unclarity in transcripts (n=4, 20%). Of the remaining 37 transcripts, 35 (94.6%) adhered to all 5 steps.

### Coach Experience

Three lay coaches and one head coach participated in the focus group discussion. Overall, coaches reported that the method and application exceeded their initial expectations, particularly in terms of ease of use and perceived user engagement. Most had no prior experience with comparable digital mental health platforms and had anticipated greater technical challenges and lower engagement.

Lay coaches reported that the initial training was helpful in introducing the Inuka method and digital platform, particularly given their limited prior experience in mental health support. However, they described the training as largely theoretical and expressed a need for more context-specific examples, practical case studies, and structured follow-up training. These needs became more pronounced as case complexity increased following the escalation of violence after October 7, 2023.

Coaches also recommended establishing peer support groups to facilitate experience sharing and collective problem-solving. While they were generally satisfied with Inuka’s technical support, they emphasized the need for more formalized technical, clinical, and psychosocial support structures to address both the demands of complex cases and the coaches’ own emotional burden after October 7, 2023.

Regarding user engagement and usability, coaches valued the platform’s structured, stepwise approach and interactive features such as real-time typing indicators, which supported rapport. They suggested several improvements, including message reactions, immediate session booking based on availability, shorter and more flexible sessions, and additional communication modes (eg, voice notes or calls), particularly after October 7, 2023. Usability challenges included the absence of an Arabic-language guide for coaches, delays in message delivery, and limitations in note-taking functionality.

In terms of accessibility, reliance on text-based communication was perceived as a barrier for individuals with low literacy, slower typing skills, or limited concentration—challenges that became more pronounced during the escalating violence.

Coaches expressed confidence in the platform’s privacy and security and reported high overall satisfaction with the program’s potential value. However, they noted that the war substantially disrupted engagement, outreach, and session continuity and that the structured session format was not always well aligned with users’ acute needs during periods of crisis.

### Adaptations During Implementation

Effective team communication was critical to the successful implementation of this activity, particularly given the volatile context characterized by continuous and often unpredictable changes. To ensure coordinated action and timely adaptation, biweekly meetings were convened with all project partners. These meetings served as platforms to review progress, identify emerging challenges, agree on necessary adjustments, and document decisions through minutes.

In addition, “dry runs” were conducted prior to full implementation. These simulation exercises allowed the team to test procedures, clarify roles and responsibilities, identify bottlenecks, and refine workflows in advance. As a result, potential challenges were addressed proactively, contributing to smoother execution and enhanced implementation fidelity during actual rollout.

Table 2 summarizes the key adaptations made during implementation of the pilot, the contextual factors that prompted these changes, and the resulting implementation learnings. It illustrates how the intervention, delivery processes, and partnerships evolved in response to operational constraints, user needs, and the escalation of violence after October 7, 2023.

**Table 2.** Adaptations during the piloting of Inuka Coaching in Palestine and key implementation learnings.

Domain	Original plan or assumption	Contextual trigger or finding	Adaptation made during implementation	Learning
Partnership and collaboration	Collaboration largely coordinated remotely	JS (KIT Institute) and AB (Inuka Coaching) work trip to Juzoor in Palestine (May 2023)	In-person meetings with Juzoor leadership, head and lay coaches, and customer support to align expectations, cocreate solutions to pressing challenges, and refine workflows during dry run	In-person meetings during project initiation are critical for trust building, clarity, and collaboration even with long-standing partners; remote-only initiation is insufficient
Training delivery	Online training for head and lay coaches	Poor connectivity, limited interaction, and difficulty asking questions	Training delivered online but identified as suboptimal; need for in-person delivery recognized	Future implementations should prioritize in-person initial training by the Inuka team, particularly for complex interventions
Coach capacity	Training focused on problem-solving methodology	Coaches reported that clients frequently needed to “vent” and sought direct advice or solutions; increased emotional burden and case complexity after October 7, 2023	Coaches adapted sessions to allow for more space for listening and emotional containment; recognition of need for additional PFA^a^ training for coaches	PFA training, structured peer support, and follow-up training are critical for lay coaches operating in conflict settings
Recruitment strategy	General outreach	Variable engagement	Recruitment diversified through universities and social media influencers; subsequent surge in demand overwhelmed staff capacity	Partnerships with educational institutions and youth networks support more sustainable recruitment; social media as part of the recruitment strategy; recruitment strategies must be matched to delivery capacity
Target population	Broad adult population envisioned in feasibility phase	Higher engagement among younger, technologically savvy users	De facto shift toward younger participants	Target population assumptions should remain flexible and be revisited during implementation
Language and framing	Intervention framed as mental health support	Stigma and hesitancy around mental health terminology	Intervention reframed as a problem-solving life skills program	For new interventions, culturally sensitive framing is critical in the early phases of implementation before trust with users has been established
Content focus	Psychosocial problem-solving	Escalation of violence after October 7, 2023	Sessions shifted toward practical concerns and emotional containment	Intervention content must be agile and responsive to rapidly changing needs
Intervention modality	Sessions delivered via text-based WhatsApp communication due to concerns about anonymity and internet stability	Text-based delivery increased perceived safety but reduced accessibility for users with lower digital literacy; impact of message delays on user experience particularly evident after October 7, 2023	Text-based communication optimized (see the “Platform functionality” domain below); recognition of the need for additional communication options (voice notes and calls)	Modality to be expanded in future versions
Platform functionality	Absence of a real-time typing indicator; no Arabic side panel for coaches; lack of emojis or message reactions; session notes aggregated rather than session specific; character limits for notes; mandatory 24-h delay between sessions	Reduced engagement and increased uncertainty during sessions; added cognitive burden for coaches due to real-time translation; omission of relevant information because of note length restrictions; limited flexibility for timely follow-up	Typing indicator added during implementation; supporting phrases and questions translated into Arabic and integrated into the platform	Simple technical adaptations can meaningfully improve usability, engagement, and perceived responsiveness
Session structure	Fixed session length and structured steps	Acute distress, particularly after October 7, 2023	Informal flexibility in pacing and session focus adopted by coaches	Rigid structures may be poorly suited to crisis contexts; flexible session formats are needed
Retention	Regular follow-up anticipated	Escalation of violence after October 7, 2023	Participants in Gaza lost to follow-up; delivery disrupted	Attrition reflects contextual constraints rather than intervention acceptability
Organizational capacity	Implementation accounted for an unstable context and relied on an experienced humanitarian NGO^b^	Scale and intensity of violence following October 7, 2023, far exceeded anticipated scenarios; death of Juzoor staff in Gaza; emergency reprioritization	NGO priorities shifted to emergency response	Implementation timelines and expectations must account for humanitarian shocks
Funding and timelines	Fixed project timeline	Implementation delays due to conflict	Funding extension granted	Even greater flexibility in funding structures would support implementation in volatile contexts

a PFA: psychological first aid.

b NGO: nongovernmental organization.

## Discussion

### Main Findings

This case report describes the implementation of a task-shifted digital mental health intervention in the humanitarian context of the West Bank and Gaza and documents how the intervention, study protocol, and partnerships evolved in response to rapidly changing circumstances. While the pilot was initially designed to explore preliminary effectiveness and acceptability, the escalation of violence after October 7, 2023, fundamentally altered the conditions under which the intervention was delivered. As such, the primary contribution of this study lies not in outcome evaluation but in the documentation of implementation challenges, adaptations, and lessons learned during a period of profound disruption.

Key implementation learnings include the importance of early in-person collaboration and training; culturally sensitive framing to support acceptability; flexibility in session content, delivery, and timelines; and the need for robust training and support structures for lay coaches. Despite such adaptations, the magnitude and rapid escalation of events—including internet outages, movement restrictions, and the death of staff within the implementing organization—exceeded anticipated scenarios and, ultimately, constrained recruitment, retention, and delivery capacity. Our case report thus illustrates that, while adaptability is essential, there are limits to what program-level adjustments can achieve in the face of large-scale humanitarian shocks.

### Limitations of Digital Collaborations

Despite a long-standing partnership between the organizations and the authors, this case demonstrated that early, in-person engagement remains critical when initiating new interventions in complex settings. Although trust, role clarity, and shared ownership were already established, reliance on remote-only initiation proved insufficient for fully aligning expectations, identifying practical constraints, and cocreating solutions to context-specific challenges. A joint visit by the KIT Institute and Inuka Coaching to Juzoor in Palestine in May 2023 was instrumental in deepening contextual understanding and supporting meaningful engagement with lay coaches and local staff. This experience illustrates that, despite the more restrictive travel policies adopted in the wake of the COVID-19 pandemic and growing attention to sustainability, intentional face-to-face interaction at the outset of a project remains a key enabler of effective partnership, shared understanding, and successful implementation.

### Finding the Right Language

Recruitment strategies varied in effectiveness. Successful methods included partnering with universities and leveraging youth coaches to promote the platform. Traditional outreach methods such as word of mouth were less effective, and social media engagement—though most effective—required continuous effort. Recruitment through influencers led to a sudden increase in engagement but overwhelmed staff capacity, highlighting the need for balanced and scalable approaches. Although the implementing organization, Juzoor, is a well-trusted actor in the field of mental health, introducing a new intervention still required careful, culturally sensitive framing, particularly during the early phases of implementation, before trust with users had been established. We found that positioning the intervention as a problem-solving life skills program as opposed to a mental health intervention appeared to lower stigma-related barriers and align more closely with participants’ preference for practical, skill-based support.

### Task Shifting Is Viable but Requires Strong Support Structures

Lay coaches played a central role in navigating the tension between the structured problem-solving model of Inuka and the realities of supporting individuals amid a genocide. Coaches reported that participants frequently needed to “vent” and sought direct advice or solutions, placing emotional and ethical demands on coaches that extended beyond the original scope of training. While the train-the-trainer model supported fidelity to core intervention steps, lay coaches voiced a desire for more flexibility and autonomy. They also highlighted the need for additional competencies, including psychological first aid skills, clearer guidance on boundaries and advice giving, structured peer support, and ongoing supervision. These findings echo feedback from lay health workers deploying the Friendship Bench in Zimbabwe [5] and reinforce that task-shifting approaches require robust and sustained support structures to safeguard both intervention quality and coach well-being [19].

### Attrition Reflects Contextual Constraints Rather Than Intervention Acceptability

Despite iterative adaptations, and in line with e–mental health interventions in other settings [10,20], this pilot was unable to prevent substantial attrition. Many participants disengaged before completing a first or second session, which may reflect competing priorities, disruptions related to violence, or the psychological burden of living in a volatile environment. In contrast, participants who progressed beyond the first sessions were more likely to complete the full program. While early disengagement may reflect contextual constraints rather than shortcomings of the intervention itself, we hypothesize that, in our context, it probably signaled misalignment among recruitment strategies, intervention design, and participants’ capacity to engage during a crisis. Future interventions in similar settings may benefit from lower-commitment models, such as single-session or brief interventions, asynchronous content delivery, or flexible pacing that accommodates interruption and uncertainty.

### Strengths and Limitations

Strengths of this study include its implementation in a country exposed to multiple crises; the adaptation of interventions to the local context; and the successful training of head coaches, lay coaches, and customer service personnel. Having the local nongovernmental organization Juzoor be in the lead was also a key strength, enhancing the relevance and acceptance of the intervention. Collaboration among the 3 implementing partners was good and maintained throughout a war, demonstrating ownership. Recognizing the need for mental health support and the added value of the piloted approach, Juzoor continued to integrate Inuka Coaching into their other programs after the pilot was done.

However, this study faced several limitations, including the small sample size, low retention, and lack of long-term follow-up data. The highly volatile context posed challenges to consistent delivery and evaluation of the intervention. Additionally, limiting interactions to written text excluded participants with low digital literacy, and our study did not investigate user experience from the participants’ perspective. Participants were not restricted from accessing other forms of support, and concurrent service use was not systematically measured. Future studies should focus on addressing these limitations to enhance the scalability and sustainability of digital mental health solutions in conflict-affected settings. Improving community involvement, refining recruitment strategies, and further contextualizing the digital platform are essential steps for future implementation efforts.

### Conclusions

Adaptability is essential but not sufficient when implementing task-shifted digital mental health interventions during acute humanitarian crises. The implementation of our pilot in Palestine was constrained not by a lack of commitment or innovation but by the scale and severity of events that exceeded anticipated scenarios. These findings underscore the importance of designing, funding, and evaluating digital mental health interventions with explicit recognition of instability, disruption, and ethical limits to engagement in conflict-affected settings.

## Supplementary material

10.2196/77507 Multimedia Appendix 1 Communication materials for the promotion of the Inuka platform.

10.2196/77507 Multimedia Appendix 2 Instagram reel: Palestinian influencer promoting the mental health digital platform.

10.2196/77507 Multimedia Appendix 3 Coach focus group discussion user guide.

## Abbreviations

CMD: common mental health disorder
KIT: Koninklijk Instituut voor de Tropen
PCL-5: Posttraumatic Stress Disorder Checklist for the Diagnostic and Statistical Manual of Mental Disorders, Fifth Edition
PTSD: posttraumatic stress disorder
SRQ-20: Self-Reporting Questionnaire–20
