# Supplementary material for: Piloting a Digital App Based on the Friendship Bench for Depression and Anxiety in Palestine: Mixed Methods Study
Source: JMIR Ment Health. 2026 Aug 5;13:e77507. doi: 10.2196/77507 (PMC13441113; doi:10.2196/77507)
Supplement: Supplementary file 1 [file mental-v13-e77507-s001.docx]

**Appendix 2**

Communication materials for the promotion of the Inuka platform.

The first set of materials focused on anxiety and depression and included a large amount of information; however, the language could be perceived as stigmatizing. Recognizing these pitfalls, the team developed a second set of marketing images using simpler, more accessible language that focused on everyday challenges people may experience, such as difficulties with time management, loneliness, and sadness. The second set of materials generated stronger engagement, suggesting greater resonance with participants.

**Set 1:**

**Set 2:**
