# Supplementary material for: Piloting a Digital App Based on the Friendship Bench for Depression and Anxiety in Palestine: Mixed Methods Study
Source: JMIR Ment Health. 2026 Aug 5;13:e77507. doi: 10.2196/77507 (PMC13441113; doi:10.2196/77507)
Supplement: Supplementary file 2 [file mental-v13-e77507-s003.docx]

**Appendix 4**

Focus Group Discussion: Topic guide for coaches’ user experience

1. What was your main expectation when starting to use the Inuka? [baseline]
2. Do you (or did you in the past) have experience with other apps, websites and resources for the same purpose as the Inuka? [baseline]
3. How was your training with Inuka as a coach? [training feedback]
4. What do you like about the Inuka? [analytical categories]
5. What don’t you like about the Inuka? [analytical categories]
6. Did you feel stuck at any moment when using the app? If yes, tell what happened. [analytical categories]
7. What improvements could be made to make the Inuka app easier or better to use? [analytical categories]
8. What haven’t we asked you today that you think would be valuable for us to know? [analytical categories]
9. What was the impact of the current war on your use or experience of the app? [analytical categories]
10. May I contact you if we have any other questions or for possible further research for this project?
